# Notes and News

**Published:** 1888-03-17

**Authors:** 


### Notes and News.

Collected and Communicated.

Lord Randolph Churchill will take the chair at the annual festival dinner of St. Mary's Hospital, which is fixed to take place on Saturday, May 12th. We hope this example may be followed by all the other metropolitan Members who ought to display an equal interest in the walfare of the hospitals of their constituences.

The amount of money bequeathed in large sums for charitable purposes during last year was over £700,000. This estimate takes no account of bequests under £1,000. If these were added, the total would more than reach £1,000,000.

The Queen, in commemoration of her recent visit to the Royal National Hospital for Consumption, at Ventnor, has sent to the authorities four large etchings of herself, the Prince Consort, the Duke of Albany, and Princess Beatrice to adorn the dining-hall.

The Bishop of Ripon, preaching in St. Paul's Church Leeds, on Hospital Sunday (the 19th ult.), expressed his regret that the collections for the hospital had been falling off during the last two years. He said that no class of men had shown themselves so wondrously self-denying in philanthropy, so entirely unselfish, or so heroic in their ministry to their fellow-men as the doctors of England; and it ought to put the public to shame if they did not adequately support the hospitals which these men tended and cared for with so much devotion and heroism.

At the annual court of Governors of the Berkshire Hospital two suggestions were made which prove that those interested in that institution are somewhat exacting. There is at present a vacancy in the medical staff of the hospital, owing to the death of Dr. Shea, and the first suggestion came in the form of a memorial praying the governors not to appoint a general practitioner to the post, but to choose a gentleman who practised only as a physician. The idea of the memorialists was to have a doctor who would act as a consultant when a second opinion was wanted in any case. This might do very well for the medical department, but what was to happen when the case was a surgical one the memorialists do not seem to have asked themselves. The second suggestion was that not more than one member of a medical firm should be appointed to the hospital staff; but as such a rule might, on occasion, prevent the hospital from gaining the services—purely honorary and gratuitous, be it understood—of really capable doctors, the governors wisely refused to consider the idea, and rejected both counsels.

At the annual meeting of subscribers to Leek Cottage Hospital a discussion arose regarding a rule which declares that persons who have been in receipt of parochial relief within six months are not eligible for admission into the hospital. Some of those present protested against the rule as bearing hardly on poor people who were struggling to get out of the condition of paupers; while others held that it was necessary to prevent the guardians evading their duty of looking after the poor, and casting their burdens on the hospital. Perhaps the matter might be compromised by the rule being abandoned and the guardians becoming subscribers to the hospital as they have done at Bristol.

The question of provident dispensaries is being canvassed very severely in Birmingham at present, and the condition of affairs there seems to be eminently unsatisfactory. Mr. Edward L. Freer, M.R.C.S., says that the majority of the members of these dispensaries are first, those who are so poor that they would be better off with parish attendance, the amount of the small subscription they paid to the dispensary being of much more use if expended in purchasing little luxuries in addition to what relief they might obtain from the parish; and second, persons in a fairly good position who ought to be the private patients of the medical men in the neighbourhood. Between the two classes the payment afforded to the dispensary doctors was rather less than threepence a visit. This certainly cannot be called adequate remuneration for any man possessed of the education and skill required of a doctor; and as medical men as a class are so exceptionally ready to work for nothing, it is very desirable that their paying patients should give them something more than a nominal fee. One thing is certain, that the multiplication of threepence-a-visit dispensaries will result in the increase of the number of practitioners who, having obtained a diploma cheaply and easily, will give—well—just as much skill and attention as threepence a visit can demand.

At the meeting of the Stoke-on-Trent Board of Guardians it was stated that two deaths had occurred in the local Contagious Diseases Hospital from small-pox, which had been brought from Nottingham. It appears that a comic singer and his son had been fulfilling an engagement in Nottingham, and during the stay there had slept in a bed in a lodging-house which had been previously occupied by two children suffering from small-pox. The owner of the lodging-house did not inform them of this fact, nor had he had the bed disinfected, and it was not until they had occupied the bed several days that they became acquainted with the terrible risk they had run. On finishing their engagement in Nottingham, they entered into another engagement at Longton, where, after performing a few nights, the boy was seized with the disease, and a few days afterwards another man in Longton also fell a victim to it. Both cases were removed to the Contagious Diseases Hospital, where they have just died. The clerk of the Stoke guardians, learning of the gross neglect of the Nottingham lodging-house keeper, and suspecting that he might not have informed the local authorities there of the existence of the disease in his house, wrote to the Town Clerk of that town, and from a communication he received from the Town Clerk he found that his suspicions were correct, and that since the outbreak of the disease in his house the man had carried on his business as usual, and other lodgers had slept in the same bed that the unfortunate children had just occupied. Until the notification of infectious diseases is much more rigorously insisted upon than it is at present, and any neglect of it is made penal, we cannot expect to escape from cases like this, which endanger the lives not only of isolated individuals, but of whole communities, by allowing the spread of infection from one town to another, without possibility of check.

THE following vacancies are announced this week, the amount of salary in each case being given after the name of the institution :—

**Resident Medical Officers.**

British Seamen's Hospital, Cronstadt, St. Petersburg.—£180 per annum, with furnished apartments.
Coton Hill Lunatic Asylum Assistant Medical Officer.—£100 per annum, with board
East London Hospital for Children, Shadwell, E. Resident Clinical Assistant.- Board; no salary.
Hospital for Consumption and Diseases of the Chest, Brompton. Resident Clinical Assistants.
Leicester Infirmary and Fever House. Assistant House Surgeon. £50 per annum, with board.
Liverpool Dispensaries. Two Assistant Surgeons.—£80 per annum, with board.
Ross-shire, Parish of Resolis and District. Resident Medical Officer.—£62 per annum.
St. Helen's Friendly Societies' Medical Aid Association. Resident Medical Officer.

**Non-resident Medical Officers.**

City of Aberdeen. Medical Officer of Health.—£300 per annum.
Glenmuick Parochial Board. Medical Officer.—£45 per annum.
Ludlow Union. Medical Officer, Munslow District.—£70 per annum, and fees.
West London Hospital, Hammersmith Road. Two Clinical Assistants.

**Matron.**

Wrexham Infirmary. Lady Matron.

**Nurses.**

Victoria Hospital Home, Margate. One, Trained.
Nursing Institute, Ryde, Isle of Wight. Three, Medical and Surgical, Midwife, and General.
Monkwearmouth Dispensary, Sunderland. One, Trained Surgical. —£16.
Kingston Nurses' Home, Baker Street, Hull. Several, Monthly, Surgical, and Massage.
Workhouse Infirmary Association, 6, Adam Street, Adelphi. Several.
Lowestoft Hospital. One.—£20.
Victoria Hospital Convalescent Home, Margate. One, Trained.
Hospital for Gentlewomen, 90, Harley Street. One, Trained. —£25.

**Probationers.**

The Cottage Hospital, Llandudno. One.
Bristol Hospital for Sick Children and Women. Several.

---

A MEETING has been held in the Infirmary Offices, Fawcett Street, Sunderland, to consider ways and means by which £2,000 may be raised for the purpose of clearing the Infirmary of debt. A four days' bazaar, to be held in the Artillery Drill Hall, is suggested as a suitable method.

MR. W. HEPPER, the executor of the late Mr. Henry Quinn, has apportioned £5,000 of this bequest to the charities of London and neighbourhood, in aid of the building fund of the Great Northern Central Hospital, on condition that a ward in the new hospital at Holloway be named the "Henry Quinn" ward, after the testator.

---

THE trustees of the Greenock Infirmary held their annual meeting, to mourn, as other hospitals are doing, over an expenditure considerably in excess of their income. This is due in great part to the lack of legacies during last year; but legacies are not a source of income on which any institution can rely. Mr. Nicol said there was room for still further economy in the working of the infirmary; but Councillor Rodger, while promising on behalf of the directors that all possible care would be taken of the funds, pleaded that the deficit at Greenock was not greater in proportion than that of the Western Infirmary at Glasgow. Still, £1,012 is a large balance to have on the wrong side of the bank-book, even for an institution which treats 953 patients annually. We agree with Mr. D. O. Leitch, who deprecated the current notion that unless a person could subscribe a guinea to the infirmary he should not subscribe at all. Every shilling, every penny, helps the total. The hospitals who have many donors of small sums are usually the most prosperous.

IT is proposed to amalgamate the Lawn Hospital at Mansfield and the Mansfield Woodhouse District Hospital.

---

THE churchwardens of Bexley have transferred to the Cottage Hospital the sum of £31 17*s.* 6*d.*, being the balance of a soup kitchen fund raised some years ago.

---

MR. ROBERT RENTOUL thinks that dentistry is neglected in the hospitals of Liverpool; out of seventeen medical charities, eleven, he says, are above taking notice of people's teeth. Considering how much agony people suffer from these necessary but troublesome members, it is not a little remarkable that at least one dentist is not attached to the staff of every hospital.

---

CONSIDERABLE discussion is going on in Belfast as to the advisability of building a hospital for consumption. As the best efforts of the committee appointed for the purpose have failed to raise the sum necessary to secure Mr. Forster Green's conditional gift, we may presume that the general feeling of the community is expressed in Mr. George Fisher's letter to the *Belfast News-Letter*, in which he expresses the opinion that the money would be better spent in providing for the comfort of patients at their own homes. There is a great deal to be said for this notion, and as the treatment of consumption depends so much on climate, the charitable public might do much good by providing funds which would enable consumptive patients to go to hospital-homes in suitable localities.

---

THE Richmond Hospital, which began last year with a deficit of £125 19s. 1d., shows at the end a surplus of £163 17s. 5d. This gratifying result is, however, due to exceptional circumstances—two large legacies, and a donation of a hundred guineas from Miss S. R. Brumly, besides Jubilee donations from Kingston-on-Thames and Richmond, and a special collection towards the deficit of 1886. An increasing source of income is found in the collecting boxes, which largely represent the offerings of the working classes, and which last year realised £313 11s. 7d. A Convalescent and Surgical Aid Fund has been formed as a memorial of the late Mr. Frederick Chapman, the subscriptions towards which amount now to £259 2s. 6d. At the last annual meeting, held at the hospital on February 28th—Admiral Stopford being in the chair—Sir Francis Burdett, Bart., resigned the post he has held for many years of auditor of the hospital accounts.

AT the forty-seventh annual meeting of subscribers to the Queen's Hospital, Birmingham, the Chairman (Lord Leigh) alluded to the Jubilee address presented by the officials and patients of the hospital to her Majesty, whose interest in the institution was proved by her donation of £100. The Rev. J. C. Blissard said that the financial position of the hospital was more satisfactory than it had ever before been, although the number of patients had increased at a rate which might cause dismay to the managers. At the Queen's Hospital the increase during the last five years had been about 60 per cent.; the number of in and out patients treated there last year being about 29,000. He expressed a hope that the funds would be sustained in such a manner as to meet the demands upon the hospital accommodation. The general opinion of the meeting seemed to be in favour of the introduction of the ticket system, as tending to make subscribers more practically interested in the working of the hospital, and also preventing those profiting by the gratuitous aid of the hospital who were able to pay for medical attendance. The Mayor (Councillor M. Pollack) moved a resolution offering the cordial thanks of the meeting to the working men of Birmingham for their donation of £1,115 19*s.* 10*d.*, and also to the contributors to the Hospital Sunday Fund, from which the Queen's Hospital received £4,181 last year.

